# Symptoms of Allodynia and Pain Thresholds Amongst Those with Acute Post-Traumatic Headache Attributed to Mild Traumatic Brain Injury: A Prospective, Longitudinal Study

**DOI:** 10.21203/rs.3.rs-7511029/v1

**Published:** 2025-09-09

**Authors:** Noah Ziff, Gina Dumkrieger, Santiago Garza, Amaal Starling, Dmitry Esterov, Kevin M. Barrett, Katherine Ross, Trent Anderson, Frank Porreca, Edita Navratilova, Simona Nikolova, Jing Li, Teresa Wu, Matthew Huentelman, Catherine D. Chong, Todd J. Schwedt

**Affiliations:** Mayo Clinic; Mayo Clinic; Mayo Clinic; Mayo Clinic; Mayo Clinic; Mayo Clinic; Phoenix VA Health Care System; University of Arizona; University of Arizona; University of Arizona; Mayo Clinic; Georgia Institute of Technology; Arizona State University; Translational Genomics Research Institute; Mayo Clinic; Mayo Clinic

**Keywords:** post-traumatic headache, concussion, traumatic brain injury, allodynia, quantitative sensory testing

## Abstract

**Background:**

Post-traumatic headache (PTH) is a common acute and persistent symptom following mild traumatic brain injury (mTBI). Symptoms of cutaneous allodynia and presence of nociceptive sensitization might be associated with acute PTH and its persistence. The objectives of this study were to compare allodynia symptoms and cutaneous heat pain thresholds amongst males and females with acute PTH to healthy controls (HC) and determine if pain thresholds and allodynia symptoms are associated with PTH outcomes.

**Methods:**

This prospective longitudinal study enrolled 139 adults with acute PTH attributed to mTBI as defined by the International Classification of Headache Disorders and 95 HC. All PTH participants completed a baseline research visit near PTH onset and a follow-up visit three to four months later. All PTH participants and a subset of HC completed the Allodynia Symptom Checklist (ASC-12) at each research visit. A different subset of the participants underwent quantitative sensory testing (QST) during baseline, 4-week, and 16-week research visits to quantify cutaneous heat pain thresholds at the forehead and forearms. Data from daily headache diaries were used to determine longitudinal PTH improvement versus non-improvement at three months. ASC-12 score and pain threshold comparisons were made between PTH and HC groups, PTH improved versus non-improved cohorts, and between PTH males and females.

**Results:**

Participants with PTH had an average age of 42.6 years and 64.0% were female. HC had an average age of 40.0 years and 65.3% were female. At the first visit, PTH participant ASC-12 scores averaged 3.6 versus 0.1 amongst HC, p < 0.001. 44.8% of PTH participants had headache improvement at 3 months. ASC-12 scores were higher in the PTH non-improved versus improved group at baseline (4.0 versus 2.4, p = 0.038) and 3-month follow-up (3.4 versus 1.9, p = 0.012). ASC-12 scores were higher in females than males at baseline (4.7 versus 1.6, p < 0.001) and 3-months (3.9 versus 1.2, p < 0.001). Cutaneous heat pain thresholds at the forehead and forearm did not differ between any group.

**Conclusions:**

PTH attributed to mTBI is associated with symptoms of cutaneous allodynia. Greater allodynia symptoms are present in females with PTH compared to males and may be associated with PTH non-improvement.

## Background

Traumatic brain injury (TBI) is a common occurrence, resulting in over 2.4 million emergency department visits each year in the United States alone.^1^ Mild TBI (mTBI) is the most frequent severity subtype, accounting for approximately 79% of all TBI cases.^2^ Post-traumatic headache (PTH) is the most common persistent symptom post-mTBI.^3^ PTH, whether acute or persistent, can be a debilitating condition, causing sequelae such as anxiety, depression, significant pain, and reduced functioning.^4^

Approximately 30–50% of acute PTH continues for at least three months and is therefore classified as “persistent” PTH.^5–7^ Understanding the mechanisms for and clinical predictors of PTH persistence could facilitate interventions that reduce the risk of PTH persistence. One such mechanism that is often implicated in the chronification of pain conditions is the development of peripheral and central sensitization. Amongst other potential contributing factors, central nervous system inflammation and alterations in cerebral metabolism that occur following mTBI are likely to contribute to the development and maintenance of sensitization amongst those with PTH.^8, 9^

Prior work demonstrates potential sex-differences in the magnitude and persistence of symptoms following mTBI, including PTH persistence, and in mechanisms that could contribute to such persistence.^10–12^ Several, but not all, studies have identified female sex as a risk factor for developing PTH and PTH persistence.^11–14^ There are numerous potential explanations for sex-specific differences in mTBI and PTH outcomes, one of which is differences in development of sensitization and allodynia. Several studies demonstrated that females are possibly more likely to develop sensitization and allodynia compared to males, perhaps partially due to the impacts of female sex hormones such as prolactin and a differential impact of calcitonin gene-related peptide (CGRP) on females compared to males.^15–17^

Prior research has shown somatosensory dysfunction in patients with PTH^18^ and identified the presence of allodynia amongst those with PTH and mTBI.^19^ Our research reported herein expands upon those findings by longitudinally investigating symptoms of allodynia and cutaneous heat pain thresholds amongst those with PTH and healthy controls (HC), investigating associations between these measures with PTH outcomes, and interrogating sex differences. We hypothesized that 1) those with acute PTH would have lower heat pain thresholds and more symptoms of cutaneous allodynia compared to HC, 2) amongst those with PTH, lower heat pain thresholds and greater allodynia would be associated with PTH persistence, and 3) females would have greater allodynia than males.

## Methods

Participants included in this analysis were enrolled into one of two studies with similar methodology. Both were prospective longitudinal studies that enrolled individuals with acute PTH and HC. Institutional Review Board approvals for the studies were obtained from the Mayo Clinic, Phoenix Veterans Administration (VA) Health Care System, and the United States Department of Defense Human Research Protection Office. Individuals with PTH were identified from the Mayo Clinic emergency room, concussion clinic, and headache clinic, as well as from the Phoenix VA emergency room and clinics. All participants completed an informed consent process and signed informed consent forms. Enrollment took place from 2019 to 2025. All PTH participants and a subset of HC completed the Allodynia Symptom Checklist (ASC-12), and a subset of participants who were studied at the Mayo Clinic Arizona underwent quantitative sensory testing (QST) for measurement of heat pain thresholds.

Participants were 18 to 70 years old at the time of enrollment. PTH participants had acute PTH attributed to mTBI as defined by the International Classification of Headache Disorders, 3rd edition diagnostic criteria,^7^ had PTH onset 0–59 days prior to enrollment, and were willing and able to maintain a headache diary and return for follow-up visits. Exclusion criteria included but were not limited to: use of opioids or barbiturates on an average of at least 4 days per month during the 6 months before screening; pregnancy; and positive neuroimaging findings that indicate a moderate or severe TBI. HC were enrolled from the Phoenix community via advertisements and word of mouth. HC had no history of TBI or migraine. HC could experience tension-type headaches on 3 days per month or less.

During the first research visit (i.e., baseline) and a follow-up research visit 3–4 months later, participants completed questionnaires including the ASC-12. A subset of participants (those enrolled into one of the two studies from which data were generated) also had QST completed during these two visits and during a 4-week post-baseline visit. PTH participants provided data in an electronic headache diary starting on the day of their baseline visit and continuing through their follow-up visit.

### Questionnaires

All participants completed a comprehensive set of questionnaires consistent with recommendations from the National Institutes of Health (NIH) National Institute of Neurological Disorders and Stroke (NINDS) Common Data Elements (CDEs) for headache and TBI.^20^ These questionnaires collected information about the current and any prior TBIs, post-TBI symptoms (22-item Symptom Evaluation Checklist from the Sport Concussion Assessment Tool (SCAT)),^21^ headache impact (Headache Impact Test (HIT-6)),^22^ symptoms of allodynia (Allodynia Symptom Checklist (ASC-12)),^23^ anxiety (State Trait Anxiety Inventory (STAI)),^24^ depression (Beck Depression Inventory (BDI)),^25^ and demographics.

The ASC-12 checklist quantifies cutaneous allodynia through a graded response system. It consists of 12 questions that ask how often individuals experience “increased pain or an unpleasant sensation” during common events such as combing hair or wearing eyeglasses. Participants can respond “does not apply to me, never, rarely, less than half the time, or half the time or more.” These answer choices correspond to numerical scores (0, 1, or 2) and are summed to classify allodynia as none (score 0–2), mild (score 3–5), moderate (score 6–8), or severe (score 9 or more).^23^ Participants completed the ASC-12 checklist at each research visit.

### Daily Diary and PTH Outcome

Participants completed a daily electronic diary for 3 to 4 months following the baseline research visit. In each entry, participants recorded whether they did or did not experience a headache that day, the severity of their pain, headache duration, level of functional disability due to the headache, and medication use.

PTH outcomes were determined using data from the daily headache diaries. PTH outcomes were determined only for those participants (n = 105) who provided diary data on at least 80% of days. To be classified as improved, participants must have had 50% or greater reduction in excess headaches in the third month compared to the first month; or had less than 2.5 excess headaches in the third month.^26^ Excess headaches are defined as the number of headaches an individual is experiencing above what they experienced prior to their mTBI. If neither of these criteria were met, the participant was considered to have non-improved PTH.

### Quantitative Sensory Testing

Quantitative sensory testing (QST) was used to determine cutaneous forehead and forearm heat pain thresholds. “Pain threshold” was defined as the first moment the participant began to feel pain from heat stimulation. Standardized instructions were delivered to participants prior to testing. Equipment: Medoc Pathway with 30mm × 30mm thermode. Pain threshold testing was performed on four body locations: right and left forehead, right and left ventral medial forearm. The procedure for each participant was: 1) participant adjusted to room temperature; 2) pain thresholds determined using method of limits with increase in thermode temperature of 1°C per second; 3) participant pushed button on response unit when pain threshold was reached; 4) heating process stopped immediately and thermode returned to baseline temperature. Testing was done three times in each body location and the mean of the three trials defined the pain threshold. The threshold testing order was the same for every participant: left ventral medial forearm, right ventral medial forearm, left forehead, then right forehead. Heat pain threshold testing was performed using methods shown to have high repeatability.^27^ QST was performed at three research visits: baseline, 4 weeks, and 16 weeks.

### Data Handling

Data were entered and maintained through Research Electronic Data Capture (REDCap).^28^ REDCap is a secure, web-based application used for designing and managing clinical research databases.

### Data Analysis

Chi-squared tests and Mann-Whitney U tests were used to compare the demographics and clinical characteristics of the PTH and HC groups, the PTH improved and PTH non-improved cohorts, as well as the PTH male and female cohorts. Normality distributions of the continuous data were assessed with Shapiro-Wilk tests. Chi-squared test was used to test categorical variables, whereas Mann-Whitney U test was used to test continuous variables. One-sided analysis was used to test the hypotheses that ASC-12 scores were higher in PTH versus HC, PTH non-improved versus PTH improved, and females versus males. One-sided analysis was also used to test the hypotheses that pain thresholds were lower in PTH versus HC and PTH non-improved versus PTH improved. All statistical analyses tests were performed with the use of R (R Core Team (2025). R: A Language and Environment for Statistical Computing. R Foundation for Statistical Computing, Vienna, Austria. https://www.R-project.org/). All statistical analyses used a significance criterion of p < 0.05.

## Results

### Allodynia Symptoms (ASC-12 Scores)

Analysis of ASC-12 scores included 139 participants with PTH and 59 HC. There were no significant differences in age, sex, or race between the PTH and HC groups. PTH participants were enrolled at an average of 32.0 (15.1) days post-mTBI. The TBI mechanisms in PTH participants consisted of 38.8% falls, 24.5% motor vehicle accidents, 7.2% sport-related injuries, and 29.5% other. A majority (75.9%) of PTH participants had migraine-like PTH phenotypes and 25.1% had tension-type-like PTH phenotypes (Table 1). Of the PTH participants, we had data sufficient to classify 105 as either PTH “improved” or “non-improved.” There were 47 improved and 58 non-improved participants, and there were no significant differences in age, sex, or race between the two cohorts (Table 2).

Those with PTH had significantly higher ASC-12 scores compared to HC at the baseline visit, 3.6 (3.7) versus 0.1 (0.4), p < 0.001, and at the follow-up visit, 2.9 (4.2) versus 0.2 (0.7), p < 0.001). At baseline, amongst those with PTH, 12.9% had severe allodynia, 15.1% had moderate allodynia, 21.6% had mild allodynia, and 50.4% had no allodynia. At follow-up, 11.8% had severe allodynia, 12.7% had moderate allodynia, 12.7% had mild allodynia, and 62.8% had no allodynia (Table 1).

The PTH non-improved cohort had significantly higher ASC-12 scores than the PTH improved cohort at the baseline visit 4.0 (4.1) versus 2.4 (2.7), p = 0.038, and the 3-month follow-up visit, 3.4 (4.3) versus 1.9 (3.9), p = 0.012. Furthermore, the PTH non-improved cohort had a higher percentage of participants with either moderate or severe allodynia at the baseline visit than the PTH improved cohort, 32.7% versus 12.8%. This difference persisted at the 3-month follow-up visit, 29.6% versus 16.3% (Table 3).

PTH females had significantly higher ASC-12 scores than PTH males at the baseline visit, 4.7 (3.9) versus 1.6 (2.4), p < 0.001 and the 3-month follow-up visit, 3.9 (4.5) versus 1.2 (2.8), p < 0.001. A smaller proportion of females with PTH had no allodynia compared to males with PTH, 36.0% versus 64.0%. Furthermore, a larger proportion of females with PTH had moderate or severe allodynia at the baseline visit, 38.2% versus 10.0%, and the 3-month follow-up visit, 28.2% versus 10.2% (Table 4).

Within the PTH improved cohort, females had significantly higher ASC-12 scores compared to males in the same cohort at the baseline visit, 3.4 (2.7) versus 0.8 (1.7), p < 0.001 and the 3-month follow-up visit, 3.1 (4.7) versus 0.4 (1.6), p = 0.002. Compared to males, a larger proportion of PTH improved females had moderate or severe allodynia at both visits, 17.8% versus 5.3% and 25.0% versus 5.3% (Table 5).

Furthermore, females had significantly higher ASC-12 scores compared to males in the PTH non-improved cohort at both visits, 4.9 (4.3) versus 2.0 (2.8), p = 0.006, and 4.2 (4.4) versus 1.9 (3.6), p = 0.014. A larger proportion of PTH non-improved females had moderate or severe allodynia than PTH non-improved males at both visits, 42.5% versus 11.0% and 36.1% versus 16.7% (Table 5).

### Heat Pain Thresholds - QST

Analysis of pain thresholds included 37 participants with PTH and 36 HC. There were no significant differences in age, sex, or race between the PTH and HC groups. PTH participants were enrolled an average of 29.3 (13.6) days post-mTBI. The TBI mechanisms in PTH participants consisted of 43.3% falls, 16.2% motor vehicle accidents, 5.4% sport-related injuries, and 35.1% other. A majority (75.7%) of PTH participants had migraine-like PTH phenotypes and 24.3% had tension-type-like PTH phenotypes (Table 6). Although cutaneous forehead and forearm pain thresholds were numerically lower in those with PTH compared to HC at each research visit, there were no significant differences. (Table 7). There were also no significant differences in pain thresholds between the PTH improved and PTH non-improved cohorts at any of the research visits (Table 8).

## Discussion

The main findings of this study are that PTH attributed to mTBI is associated with symptoms of cutaneous allodynia, greater allodynia symptoms during the acute phase of PTH are associated with PTH non-improvement, and females with PTH have significantly greater allodynia symptoms than males. There were no significant differences in cutaneous heat pain thresholds between those with PTH and HC and no significant relationship between cutaneous heat pain thresholds with PTH outcomes.

### PTH and Cutaneous Allodynia

Consistent with our *a priori* hypothesis, those with PTH had greater symptoms of allodynia compared to healthy controls. Prior studies have reported the presence of allodynia amongst those with PTH.^29–31^ A study by Ashina et al. of participants with PTH for an average of 49.3 months at the time of evaluation, found that allodynia symptoms were severe in 6%, moderate in 17%, mild in 23%, and absent in 54%, according to ASC-12 scores.^29^ In a prior study from our research group, 56 participants with persistent PTH (PPTH) who had PTH for a median of 8.7 years had ASC-12 scores consistent with severe allodynia in 20%, moderate allodynia in 21%, mild allodynia in 25%, and no allodynia in 34%.^30^ Finally, a study by Metti et. al. of recently deployed soldiers with mTBI found that 35.2% of those with PTH had symptoms of allodynia compared to 13.2% of those with mTBI but without PTH,^31^ suggesting an association between PTH and allodynia that is not explained by the underlying mTBI alone. Since our current study enrolled participants during the acute phase of PTH and followed them through the earliest stages of persistent PTH, our ASC-12 results are not directly comparable to the results from these prior publications which enrolled participants later in the course of PPTH. Data from our study that are most comparable are the follow-up research visit ASC-12 scores from participants who did not have PTH improvement. In that group, allodynia was severe in 16.7%, moderate in 12.9%, mild in 14.8% and absent in 55.6% (Table 3), results that are relatively similar to those from these other PPTH studies.^29^ Our study adds to the existing literature by demonstrating the presence of allodynia during the acute phase of PTH.

Our study also adds to the existing literature by including longitudinal measurements of allodynia and identifying a relationship between allodynia symptoms and PTH outcomes. Those who did not have PTH improvement were more likely to have allodynia and to have higher ASC-12 scores compared to those with PTH improvement, both at baseline and at the follow-up research visit. Although prior publications have demonstrated potential predictors for PTH persistence, such as pre-TBI headaches, TBI severity, age, and others, there is a lack of knowledge about the potential contribution of allodynia symptoms for that prediction.^32, 33^ Although this study did not develop predictive models for PTH persistence based on ASC-12 scores, it demonstrated that baseline measurements of allodynia are higher in those who do not have PTH improvement compared to those who do improve. Furthermore, ASC-12 scores remained higher at the follow-up research visit amongst those without PTH improvement, suggesting that ASC-12 scores might be predictive of PTH outcomes and a biomarker for persistent PTH. It is feasible that development of sensitization during headache attacks could lead to progressive sensitization of the peripheral and central nervous system, facilitating the persistence of PTH.

A secondary finding in our study was the association between allodynia and sex. Compared to males, females had significantly higher ASC-12 scores at baseline and follow-up, regardless of PTH improvement status. Previous studies have shown that females suffer higher disease burden post-mTBI,^34, 35^ but there is a paucity of prior knowledge specifically about sex-specific differences in allodynia following mTBI or associated with PTH. More severe allodynia in female PTH participants is consistent with previous findings in studies of migraine.^36^ Furthermore, it is known that females are more likely to develop allodynia, sensitization, and chronic pain.^37, 38^ There are numerous potential contributing factors for why females experience more symptoms of allodynia, including contributions from genetics, differences in pain physiology, impacts of sex hormones, and environmental factors.^39, 40^ Although a full discussion on mechanisms of sexual dimorphism in sensitization and chronic pain is beyond the scope of this article, there are several review publications on the topic.^37, 38, 40^

### PTH and Heat Pain Thresholds

We hypothesized that 1) those with acute PTH would have lower cutaneous heat pain thresholds than HC, and 2) amongst those with PTH, lower heat pain thresholds would be associated with lack of PTH improvement. Although heat pain thresholds were numerically lower in those with PTH compared to HC, there were no significant differences. Furthermore, there were no differences in pain thresholds amongst those with PTH who had PTH improvement compared to those without improvement. A prior study of 20 participants who had PTH for an average of 46 months found those with PTH to have lower heat pain thresholds on the forearm compared to HC but no difference at the forehead.^41^ A study of 30 participants with a median of two years of PTH found that mechanical pain thresholds, tested using Von Frey filaments, were lower at the periorbital region and forearm compared to HC.^18^ A study of 15 male participants who had experienced PTH of a migraine-like phenotype for an average of 13.2 years and 13 male participants who had experienced PTH of a tension-type headache-like phenotype for an average of 12.6 years found that those with the tension-type headache-like phenotype had abnormal thermal and mechanical sensory thresholds while those with migraine-like phenotypes had normal thermal and mechanical thresholds.^19^ Overall, the results from these published studies and our study do not clearly demonstrate that there are differences in cutaneous heat pain thresholds amongst those with acute or persistent PTH. Numerous factors could contribute to variability in findings, including absence or presence of headache or other pain during testing, duration of time since last headache or until next headache, duration since onset of PTH, PTH phenotype, participant sex, emotional processing of pain, and use of medications, amongst others. Additional studies with even larger sample sizes might be needed to account for these potentially impactful variables and determine if changes in heat pain thresholds are associated with acute or persistent PTH and their outcomes. Furthermore, future PTH studies could measure heat pain thresholds and collect information about symptoms of allodynia (e.g., using the ASC-12) at the same time during a headache to investigate correlations between them.

## Conclusion

In this prospective longitudinal study, we found that acute PTH attributed to mTBI is associated with cutaneous allodynia. Females with PTH have greater allodynia than males with PTH. The presence and severity of allodynia symptoms is associated with PTH outcomes: allodynia during the acute phase of PTH is associated with a lack of PTH improvement measured three months later. This study demonstrated that PTH and PTH outcomes are not associated with significant alterations in cutaneous heat pain thresholds. Future studies could determine the value of allodynia assessment during the acute phase of PTH for predicting PTH persistence.

## Figures and Tables

**Table 1 –:** Demographics and clinical characteristics of PTH and HC groups

Variable	PTH (n = 139)	HC (n = 59)	p-value
Age, years; mean (SD)	42.6 (16.0)	40.4 (13.0)	0.433^a^
Sex, n(%)			1.00^b^
Female	89 (64.0%)	38 (64.4%)	
Male	50 (36.0%)	21 (35.6%)	
Race, n (%)			0.813^b^
Asian	7 (5.0%)	1 (1.7%)	
Black/African American	4 (2.9%)	2 (3.4%)	
White/Caucasian	122 (87.8%)	54 (91.5%)	
Other	6 (4.3%)	2 (3.4%)	
TBI Mechanism, n(%)			
Fall	54 (38.8%)	--	--
Motor Vehicle Accident	34 (24.5%)	--	--
Sport	10 (7.2%)	--	--
Other	41 (29.5%)	--	--
PTH Phenotype, n(%)			
Migraine-like	104 (75.9%)	--	--
Tension-type-like	35 (25.1%)	--	--
Time From TBI to Enrollment, days (SD)	32.0 (15.1)	--	--
Allodynia (ASC-12)			
**Screening Visit; mean (SD), N**	**3.6 (3.7), 139**	**0.1 (0.4), 59**	<0.001^c^
None, n(%)	70 (50.4%)	59 (100.0%)	
Mild, n(%)	30 (21.6%)	0 (0.0%)	
Moderate, n(%)	21 (15.1%)	0 (0.0%)	
Severe, n(%)	18 (12.9%)	0 (0.0%)	
**Month 3; mean (SD), N**	**2.9 (4.2), 110**	**0.2 (0.7), 55**	<0.001^c^
None, n(%)	69 (62.8%)	53 (96.4%)	
Mild, n(%)	14 (12.7%)	2 (3.6%)	
Moderate, n(%)	14 (12.7%)	0 (0.0%)	
Severe, n(%)	13 (11.8%)	0 (0.0%)	

Abbreviations: PTH, post-traumatic headache; HC, healthy controls; SD, standard deviation; TBI, traumatic brain injury

a Two-sided Mann-Whitney U test.

b Chi-squared test.

c One-sided Mann-Whitney U test.

**Table 2 --:** Demographics of PTH improved and PTH non-improved cohorts

Variable	PTH Improved (n = 47)	PTH Non-Improved (n = 58)	p-value
Age, years; mean (SD)	45.1 (17.4)	42.5 (14.0)	0.292^a^
Sex, n(%)			0.426^b^
Female	28 (59.6%)	40 (68.9%)	
Male	19 (40.4%)	18 (31.1%)	
Race, n (%)			0.486^b^
Asian	3 (6.4%)	4 (6.9%)	
Black/African American	0 (0.0%)	2 (3.5%)	
White/Caucasian	40 (85.1%)	51 (87.9%)	
Other	4 (8.5%)	1 (1.7%)	

Abbreviations: PTH, post-traumatic headache; SD, standard deviation

a Two-sided Mann-Whitney U test.

b Chi-squared test.

**Table 3 –:** Comparison of allodynia (ASC-12) scores between PTH improved and PTH non-improved cohorts

Variable	PTH Improved	PTH Non-Improved	p-value^a^
Allodynia (ASC-12)			
**Screening Visit; mean (SD), N**	**2.4 (2.7), 47**	**4.0 (4.1), 58**	0.038
None, n(%)	28 (59.6%)	27 (46.6%)	
Mild, n(%)	13 (27.6%)	12 (20.7%)	
Moderate, n(%)	4 (8.5%)	10 (17.2%)	
Severe, n(%)	2 (4.3%)	9 (15.5%)	
**Month 3; mean (SD), N**	**1.9 (3.9), 43**	**3.4 (4.3), 54**	0.012
None, n(%)	33 (76.7%)	30 (55.6%)	
Mild, n(%)	3 (7.0%)	8 (14.8%)	
Moderate, n(%)	4 (9.3%)	7 (12.9%)	
Severe, n(%)	3 (7.0%)	9 (16.7%)	

Abbreviations: PTH, post-traumatic headache; SD, standard deviation

a One-sided Mann-Whitney U test.

**Table 4 –:** Comparison of allodynia (ASC-12) scores between all PTH males and females

Variable	PTH Male	PTH Female	p-value^a^
Allodynia (ASC-12)			
**Screening Visit; mean (SD), N**	**1.6 (2.4), 50**	**4.7 (3.9), 89**	< 0.001
None, n(%)	37 (64.0%)	32 (36.0%)	
Mild, n(%)	8 (16.0%)	23 (25.8%)	
Moderate, n(%)	4 (8.0%)	17 (19.1%)	
Severe, n(%)	1 (2.0%)	17 (19.1%)	
**Month 3; mean (SD), N**	**1.2 (2.8), 39**	**3.9 (4.5), 71**	< 0.001
None, n(%)	32 (82.1%)	41 (57.7%)	
Mild, n(%)	3 (7.7%)	10 (14.1%)	
Moderate, n(%)	2 (5.1%)	10 (14.1%)	
Severe, n(%)	2 (5.1%)	10 (14.1%)	

Abbreviations: PTH, post-traumatic headache; SD, standard deviation

a One-sided Mann-Whitney U test.

**Table 5 –:** Comparison of allodynia (ASC-12) scores between PTH improved males and females and PTH non-improved males and females

Variable	PTH Male	PTH Female	p-value^a^
Allodynia (ASC-12)			
**Screening Visit - Improved; mean (SD), N**	**0.8 (1.7), 19**	**3.4 (2.7), 28**	<0.001
None, n(%)	16 (84.2%)	12 (42.9%)	
Mild, n(%)	2 (10.5%)	11 (39.3%)	
Moderate, n(%)	1 (5.3%)	3 (10.7%)	
Severe, n(%)	0 (0.0%)	2 (7.1%)	
**Month 3 - Improved; mean (SD), N**	**0.4 (1.6), 19**	**3.1 (4.7), 24**	0.002
None, n(%)	18 (94.7%)	15 (62.5%)	
Mild, n(%)	0 (0.0%)	3 (12.5%)	
Moderate, n(%)	1 (5.3%)	3 (12.5%)	
Severe, n(%)	0 (0.0%)	3 (12.5%)	
Allodynia (ASC-12)			
**Screening Visit - Non-Improved; mean (SD), N**	**2.0 (2.8), 18**	**4.9 (4.3), 40**	0.006
None, n(%)	12 (66.7%)	15 (37.5%)	
Mild, n(%)	4 (22.3%)	8 (20.0%)	
Moderate, n(%)	1 (5.5%)	9 (22.5%)	
Severe, n(%)	1 (5.5%)	8 (20.0%)	
**Month 3 - Non-Improved; mean (SD), N**	**1.9 (3.6), 18**	**4.2 (4.4), 36**	0.014
None, n(%)	13 (72.2%)	17 (47.2%)	
Mild, n(%)	2 (11.1%)	6 (16.7%)	
Moderate, n(%)	1 (5.6%)	6 (16.7%)	
Severe, n(%)	2 (11.1%)	7 (19.4%)	

Abbreviations: PTH, post-traumatic headache; SD, standard deviation

a One-sided Mann-Whitney U test.

**Table 6 –:** Demographics of PTH and HC groups that completed QST testing

Variable	PTH (n = 37)	HC (n = 36)	p-value
Age, years; mean (SD)	41.9 (17.4)	39.4 (14.8)	0.713^a^
Sex, n(%)			0.529^b^
Female	21 (56.8%)	24 (66.7%)	
Male	16 (43.2%)	12 (33.3%)	
Race, n (%)			0.739^b^
Asian	3 (8.1%)	0 (0.0%)	
Black/African American	2 (5.4%)	0 (0.0%)	
White/Caucasian	31 (83.8%)	33 (91.7%)	
Other	1 (2.7%)	3 (8.3%)	
TBI Mechanism, n(%)			
Fall	16 (43.3%)	--	--
Motor Vehicle Accident	6 (16.2%)	--	--
Sport	2 (5.4%)	--	--
Other	13 (35.1%)	--	--
PTH Phenotype, n(%)			
Migraine	28 (75.7%)	--	--
Tension-type	9 (24.3%)	--	--
Time From TBI to Enrollment, days (SD)	29.3 (13.6)	--	--

Abbreviations: PTH, post-traumatic headache; HC, healthy controls; SD, standard deviation; TBI, traumatic brain injury

a Two-sided Mann-Whitney U test.

b Chi-squared test.

**Table 7 –:** Comparison of cutaneous heat pain thresholds of the PTH and HC groups

Variable	PTH	HC	p-value^a^
Cutaneous Forearm Heat Threshold, °C; mean (SD), N			
Screening Visit	44.6 (4.7), 37	45.3 (2.7), 36	0.665
Week 4	43.2 (4.5), 32	45.0 (1.8), 31	0.163
Week 16	43.8 (4.3), 25	45.0 (2.4), 28	0.206
Cutaneous Forehead Heat Threshold, °C; mean (SD), N			
Screening Visit	45.3 (3.8), 37	45.7 (3.0), 36	0.396
Week 4	43.2 (3.8), 32	44.6 (3.1), 31	0.071
Week 16	43.5 (4.5), 25	44.9 (3.1), 28	0.182

Abbreviations: PTH, post-traumatic headache; HC, healthy controls; SD, standard deviation

a One-sided Mann-Whitney U test

**Table 8 –:** Comparison of cutaneous heat pain thresholds of the PTH improved and PTH non-improved cohorts

Variable	PTH Improved	PTH Non-Improved	p-value^a^
Cutaneous Forearm Heat Threshold, °C; mean (SD), N			
Screening Visit	44.3 (4.8), 17	44.9 (4.7), 12	0.324
Week 4	43.2 (4.6), 17	43.1 (4.8), 12	0.405
Week 16	43.9 (4.0), 13	43.1 (4.6), 11	0.716
Cutaneous Forehead Heat Threshold, °C; mean (SD), N			
Screening Visit	45.3 (3.9), 17	45.2 (4.5), 12	0.491
Week 4	43.6 (3.7), 17	42.7 (4.1), 12	0.803
Week 16	43.1 (4.8), 13	43.5 (4.4), 11	0.367

Abbreviations: PTH, post-traumatic headache; SD, standard deviation

a One-sided Mann-Whitney U test

## Data Availability

The datasets generated and analyzed during the current study are not publicly available due to protected health information but are available from the corresponding author on reasonable request.

## List of abbreviations

PTH: Post-traumatic headache
mTBI: Mild traumatic brain injury
HC: Healthy controls
ICHD: International Classification of Headache Disorders
ASC-12: Allodynia Symptom Checklist
TBI: Traumatic brain imnjury
CGRP: Calcitonin gene-related peptide
VA: Veterans Administration
QST: Quantitative sensory testing
NIH: National Institutes of Health
NINDS: National Institute of Neurological Disorders and Stroke
CDEs: Common Data Elements
SCAT: Sport Concussion Assessment Tool
HIT-6: Headache Impact Test
STAI: State Trait Anxiety Inventory
BDI: Beck Depression Inventory
REDCap: Research Electronic Data Capture
PPTH: Persistent post-traumatic headache
